# Genomics and phenomics: Who will be the dairy cows of the future?

**DOI:** 10.3168/jdsc.2025-0872

**Published:** 2025-10-30

**Authors:** Luiz F. Brito, Allan P. Schinckel, Hinayah Rojas de Oliveira

**Affiliations:** Department of Animal Sciences, Purdue University, West Lafayette, IN 47907

## Abstract

The dairy industry has experienced unprecedented genetic progress, more than doubling milk yield over recent decades, but this has often resulted in reduced fertility, longevity, and robustness. This review addresses the question “Who will be the dairy cows of the future?” by highlighting the integration of genomics, phenomics, and advanced breeding strategies. From our perspective, future cows are expected to be healthier, more resilient, and longer-lived, with improved fertility, feed efficiency, and reduced methane emissions. Precision technologies, wearable sensors, and automated systems are providing novel phenotypes and driving selection for adaptability, welfare, and efficiency. Genomic selection, reproductive and other biological technologies, and beef-on-dairy crossbreeding are reshaping dairy breeding programs, while collaborations are critical for advancing multiple-trait evaluations and safeguarding genetic diversity. Despite Holstein breed dominance, maintaining across- and within-breed variation is essential for long-term sustainability. Ultimately, as a consequence of the wide adoption of precision technologies, more complex breeding goals, and effective breeding strategies, the dairy cow of the future will balance productive efficiency with resilience, welfare, and environmental efficiency, ensuring global sustainability of dairy production.

## Highlights

• Genomics and phenomics will define the dairy cow of the future.
• Future cows will be healthier, more resilient, and longer-lived.
• Precision technology enables selection for welfare and efficiency.
• Safeguarding genetic diversity is the key to sustainability.

## Graphical Abstract

**Figure:**
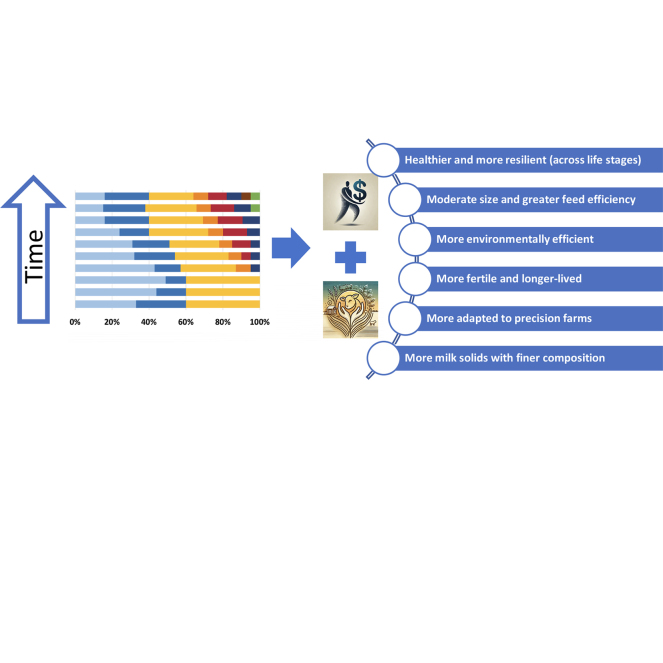
**Summary:** Future dairy cows will be healthier, longer-lived, and more fertile, with improved resilience and feed efficiency, and lower methane emissions. Advances in genomics, phenomics, and precision technologies will drive balanced genetic improvement in productivity, adaptability, welfare, and environmental sustainability. Selection indexes are being continually refined to enable coordinated genetic progress across these diverse breeding goals. Modern breeding programs will integrate genomic selection, automated phenotyping, and responsible management of genetic diversity to develop efficient and sustainable dairy populations.

The global dairy cattle industry has changed dramatically over the past century, with milk production per lactation more than doubling in recent decades (Brito et al., 2021), largely due to genetic improvement (Figure 1). However, the large increase in milk productivity per lactation has been accompanied by reduced fertility and longevity, as well as higher incidence of infectious and metabolic diseases (Lucy, 2001; Miglior et al., 2017). These negative consequences were mainly the result of an intensive and long-term focus on selection for milk production traits alone (Miglior et al., 2017). Fortunately, this trend began to shift toward the end of the last century, driven by advances in computational capacity and the implementation of dairy cattle breeding programs that directly targeted fertility, health, longevity, and other functional traits (Cole and VanRaden, 2018; Brito et al., 2021). Multiple-trait selection has become possible through major advancements in phenotyping tools and data collection systems, which, when combined with genomic selection, reproductive technologies, and improved management practices, have accelerated the rates of genetic and phenotypic change in worldwide dairy cattle populations. Dairy farms are also getting larger and labor shortages are becoming a challenge in many countries (Charlton and Kostandini, 2021). From another angle, society expects an increased and more affordable supply of milk and other dairy products made with milk from healthy cows requiring less veterinary medical treatments and being raised under exemplary welfare conditions (Barkema et al., 2015). This should be accomplished while minimizing the environmental impacts of the dairy industry. Precision technologies and wearables sensors are being increasingly used in dairy farms to optimize management, mitigate labor shortages, improve animal and farmer welfare, and generate large-scale datasets for developing novel traits and breeding goals (Brito et al., 2020a, 2025). Under these circumstances, dairy producers operate with narrow profit margins and are being required to innovate to remain competitive in the food market. In this context, the main objective of this article is to provide a perspective on the future of dairy cattle breeding by addressing the question “Who will be the dairy cows of the future?” More specifically, we discuss the key trait groups, emerging technologies, and stakeholders' decisions or actions that are shaping, or are likely to shape, the dairy cow of the future, with particular emphasis on breeding programs in developed countries.

**Figure 1:**
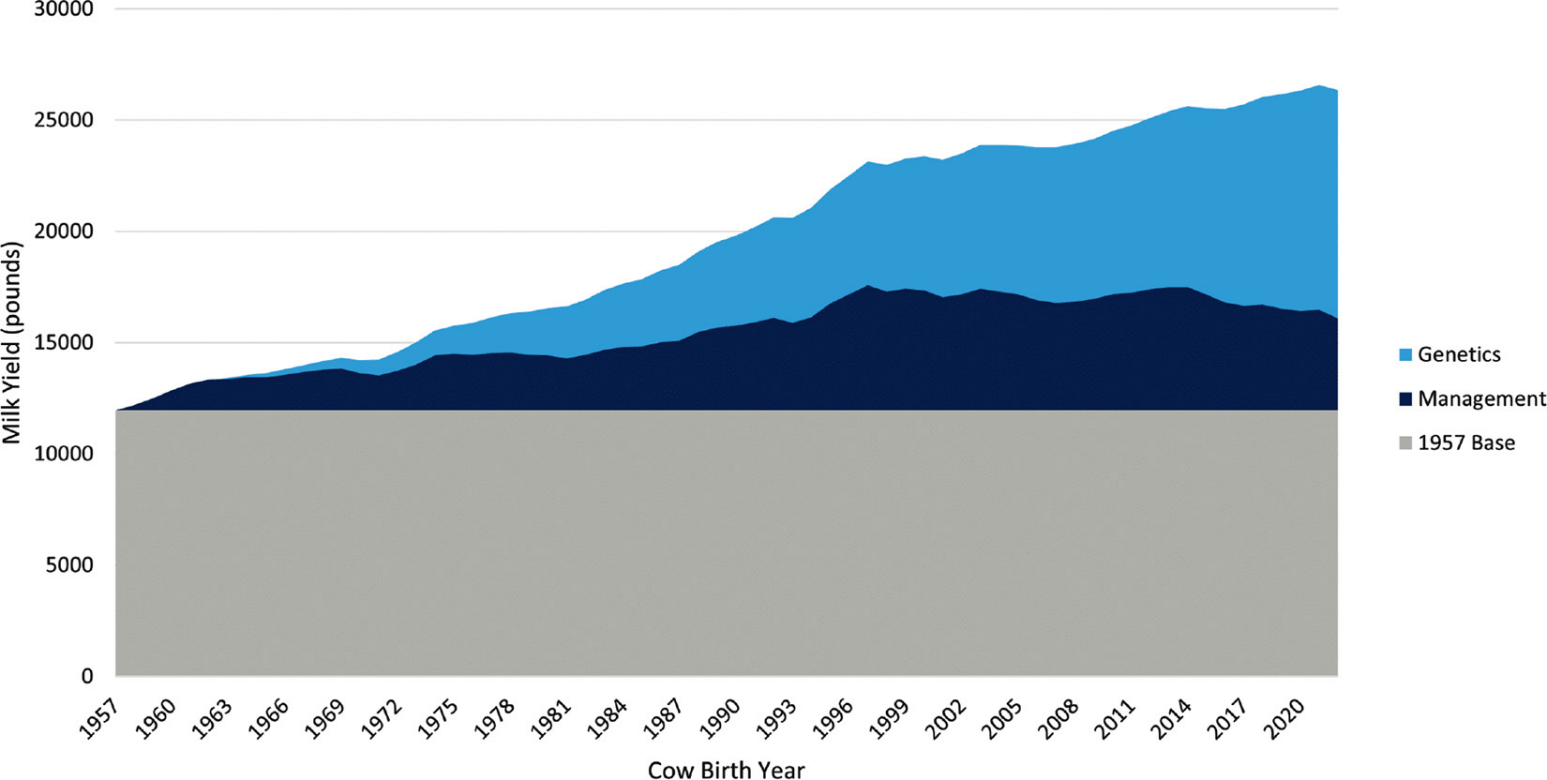
Changes in US Holstein milk production from 1957 to 2022, indicating the proportional contributions of genetics and management practices. Source: Council on Dairy Cattle Breeding (CDCB, 2025b).

Before envisioning the cow of the future, it is important to revisit past events. Following cattle domestication over 10,000 yr ago (Pitt et al., 2019), farmers began selecting breeding animals based on a limited number of traits of interest. This early domestication and selection process led to the formation of specialized breeds for distinct purposes such as meat, milk, or draft. Initially, selection relied exclusively on observable phenotypes, with little or no systematic record keeping. The primary traits targeted under artificial selection included temperament, milk yield, and physical attributes such as coat color, presence of horns, and body conformation. Despite the limited scope of phenotypic recording, substantial genetic progress was achieved for traits with moderate to high heritability such as temperament (Chang et al., 2020; Pacheco et al., 2025).

Systematic phenotypic data collection in dairy cattle populations began in the late 19th and early 20th centuries, particularly in Europe and North America, driven by the need to increase milk production and overall herd performance. Dairy Herd Information Associations with organized milk recording systems were established in the early 1900s (Voelker, 1981). For many decades, phenotypic recording and pedigree-based selection relied primarily on low-frequency measurements (e.g., monthly) of milk yield and milk composition, followed later by body conformation traits (Miglior et al., 2017). From the 1980s onward, data collection schemes expanded to include a broader range of traits related to performance, reproduction and fertility, cow longevity, health and welfare, and environmental efficiency (e.g., feed efficiency, methane emissions; Cole and VanRaden, 2018; Brito et al., 2021, 2025).

In the late 1990s, there was a need and an opportunity to develop more complex selection objectives that emphasized economic and societal importance of the individual traits simultaneously. Since then, multiple-trait selection indexes have been continually updated to reflect emerging breeding goals (Cole and VanRaden, 2018), which are contributing to shape the dairy cow of the future. Nevertheless, many traits of growing importance (e.g., health, welfare, and fertility-related traits) remain difficult to measure and generally have low heritabilities (Maskal et al., 2024). Their quantification generally requires continuous and large-scale recording, which remains a challenge. The implementation of genomic selection around 15 to 20 yr ago revolutionized dairy cattle breeding by facilitating the genetic evaluation of traits that are lowly heritable, expensive or difficult to measure, sex-limited, or expressed late in life (Brito et al., 2021; Guinan et al., 2023). Genomic selection has increased annual genetic progress by improving the accuracy of breeding values of younger animals, enhancing selection intensity, and substantially reducing the generation intervals in dairy cattle breeding programs (Guinan et al., 2023). Coupled with large-scale phenotyping, genotyping has now become routine in most major dairy populations, where reference populations comprising hundreds of thousands to millions of animals provide the foundation for highly accurate genomic predictions for many key traits. In the United States alone, more than 10 million dairy animals have been genotyped, of which ∼93% are female (CDCB, 2025a). Genomic datasets have been applied to a wide range of purposes, including genomic selection, parentage verification and correction, estimation of breed composition, culling decisions, optimization of mating schemes (e.g., identifying cows to be inseminated with beef semen in beef-on-dairy crossbreeding), and mating allocations to minimize inbreeding, among other applications. Furthermore, the identification and elimination of harmful recessive alleles has become a standard practice, with tools now routinely available to reduce the incidence of genetic defects and improve population health (Cole et al., 2025).

In addition to productive and reproductive efficiency, the dairy cow of the future must also exhibit superior health, welfare, and resilience to thrive within modern production systems and to meet society's expectations for responsible animal care. In this context, health and welfare traits are gaining prominence in breeding programs (Cole and VanRaden, 2018; Brito et al., 2020a), not only due to their direct economic impact but also in response to increasing public concern for animal well-being. Furthermore, as climate change continues to threaten livestock production, the dairy cow of the future will need to be more resilient for sustaining high performance across diverse and unpredictable environments and management conditions (Misztal et al., 2025). Resilience is defined as the capacity of animals to be minimally affected by disturbances or to rapidly recover from them (Colditz and Hine, 2016), which can be operationalized as the ability to withstand environmental stress, disease, or management challenges while maintaining productivity and recovering quickly from temporary reductions in performance. In recent years, various indicators of overall resilience have been proposed based on variability in longitudinally recorded variables such as milk yield (Poppe et al., 2020; Chen et al., 2023), activity level (Poppe et al., 2022), and calf milk consumption (Graham et al., 2024). Variance-based resilience indicators are based on the principle that more resilient animals exhibit less variation in performance measures over time. Conversely, high values of these indicators reflect lower resilience, as such animals display greater fluctuations in response to environmental challenges. Most resilience indicators proposed so far are heritable (Maskal et al., 2024; Brito et al., 2025) and can, therefore, be improved through genetic selection. A recent meta-analysis by Maskal et al. (2024) reported substantial variability in the genetic relationships between resilience and productivity traits, but provided strong evidence that simultaneous genetic improvement in both trait groups is achievable when they are jointly incorporated into selection indexes.

Intensive selection for higher milk yield has likely resulted in greater heat stress sensitivity (Boonkum et al., 2024; Misztal et al., 2025), indicating the need to breed for improved heat tolerance. The most common methods for estimating breeding values for heat tolerance largely focus on variability in production performance under thermal stress conditions, and the incorporation of genomic information has significantly improved prediction accuracy (Nguyen et al., 2016; Misztal et al., 2025). However, selecting solely for animals that maintain production during heat stress may increase mortality risk and welfare issues, highlighting the complexity of balancing breeding objectives. Therefore, there is a need for developing novel traits related to physiological, behavioral, and anatomical characteristics that may capture variability in heat tolerance more independently of milk production, thereby enabling more holistic and effective breeding for heat tolerance (Brito et al., 2025; Misztal et al., 2025). Another technology that may become highly relevant in this regard is gene editing (Van Eenennaam, 2019). Key mutations associated with heat tolerance, such as the prolactin receptor (*PRLR*) gene (“SLICK hair”), can be efficiently edited to introduce favorable alleles from more heat-tolerant cattle breeds such as Senepol into high-producing dairy breeds such as Holstein (Sonstegard et al., 2024).

The cow of the future will be healthier than today's cows. Although health traits in dairy cattle typically exhibit low to moderate heritability, they generally show substantial additive genetic variation, indicating that meaningful genetic progress can be achieved through direct selection (Zavadilová et al., 2021; Brito et al., 2025). This progress is further accelerated when genomic information is incorporated into the prediction of breeding values. Mastitis remains one of the most prevalent and costly infectious diseases in dairy cattle, and genetic selection for reduced clinical mastitis incidence, as well as for auxiliary traits such as SCS, has proven effective (Martin et al., 2018). Beyond mastitis, numerous health traits have either already been incorporated into national selection indexes or are in advanced stages of genetic evaluation and implementation. These include metabolic and digestive disorders such as ketosis and displaced abomasum, as well as lameness, hoof health, retained placenta, metritis, and respiratory diseases (Jamrozik et al., 2016a,b). There will also be a greater focus on traits related to calf health and efficiency. Continued expansion of routine phenotyping, combined with genomic prediction and high-throughput phenotyping using sensors and other technologies, will enhance the accuracy of selection for these traits and contribute to a more resilient and robust dairy cow.

Accurately measuring health and welfare traits remains challenging because many health conditions occur at relatively low frequency and their assessment or diagnosis is often subjective. Traditional recording approaches, based on producer reports or veterinary diagnoses, are often prone to incompleteness or inconsistency. To overcome these limitations, the development of objective and automated measurement systems is essential. Sensor technologies provide promising tools for continuous monitoring of health and welfare (Brito et al., 2020a, 2025). For example, activity monitors can detect behavioral changes indicative of illness or distress, whereas automated systems for recording rumination, feeding behavior, and lying patterns provide valuable insights into metabolic status and overall well-being. Moreover, the integration of multiple sensor data streams through machine learning and predictive modeling has the potential to enhance the accuracy and reliability of health and welfare assessments (Marzougui et al., 2025).

The rapid adoption of automated milking systems (**AMS**; also termed milking robots) and other precision technologies and wearable sensors in dairy farming may reshape the ideal dairy cows. The dairy cow of the future must not only possess structural soundness for longevity, efficiency, and welfare but also physical and behavioral characteristics that facilitate efficient interaction with automated systems. This represents a major shift from traditional breeding objectives, which were historically oriented toward human-operated farming. For instance, AMS rely on predictable udder characteristics for accurate teat detection and attachment as well as cow behavior. The ideal udder is characterized by appropriate height and depth, strong attachments, and optimal teat placement, length, and width. The AMS provide useful data for deriving relevant udder conformation traits based on XYZ cartesian coordinates, which are moderately to highly heritable (Medeiros et al., 2024). Milking speed is another heritable trait of high economic relevance in AMS herds. Faster-milking cows improve AMS throughput, enabling more cows to be milked per unit per day. Milking speed is a moderately heritable trait and is favorably correlated with milk yield (Pedrosa et al., 2023). Beyond milking speed, cow behavior in AMS is also highly relevant for system efficiency and animal management, and several cow behavioral traits have been shown to be heritable (Bérat et al., 2025). These include the time interval between successive milkings, the number of attempted visits to the AMS, the number of successful entries, the proportion of successful milkings, and the cow preference consistency score, all of which reflect the adaptability of cows to AMS (Bérat et al., 2025; Brito et al., 2025).

Docility, easy social interaction, and behavioral plasticity represent critical traits that influence both animal welfare and operational efficiency in modern dairy systems (Pacheco et al., 2025). Temperament, often called docility, represents one of the most important behavioral traits in dairy cattle due to its direct relationship with animal welfare, handler safety, and production efficiency. Docile cattle are easier and safer to handle, require less labor for routine management procedures, and show better adaptation to modern production systems. The economic impact of temperament extends beyond labor efficiency to include effects on milk production, reproductive performance, and longevity (Pacheco et al., 2025). Temperament traits in dairy cattle show moderate to high heritability (Chang et al., 2020; Pinto et al., 2025), depending on the specific trait definition and measurement method. These heritability values indicate that genetic improvement through selection is highly possible and can achieve substantial progress within relatively few generations.

Feed typically accounts for 50% to 70% of total production costs in dairy production systems, making feed efficiency a primary driver of economic success. Therefore, dairy cattle producers are increasingly prioritizing feed efficiency in their breeding objectives and various countries have already included it in their selection indexes (Manzanilla-Pech et al., 2022). Feed efficiency directly influences farm profitability, environmental sustainability, and the efficient use of resources. Several traits have been proposed or used for genetically improving feed efficiency in dairy cattle, including feed conversion ratio, residual feed intake (**RFI**), DMI, and efficiency ratios that consider both maintenance requirements and milk production (Tempelman and Lu, 2020). Residual feed intake is the most common feed efficiency trait in dairy cattle research and breeding programs because of its independence from production level and body size (Roche et al., 2018; Stephansen et al., 2024). This measure is calculated as the difference between actual feed intake and predicted feed intake based on BW and milk production (Tempelman and Lu, 2020; Stephansen et al., 2024). Animals with negative RFI values are more efficient because they eat less feed than predicted for their level of production. In addition to within-animal efficiency traits, broader indicators of overall cow efficiency are increasingly relevant for both management and breeding purposes, including milk solids yield relative to BW or ECM per unit of metabolic weight. These measures, although less precise in isolating biological efficiency, are easier to record on a large scale and often align closely with farm profitability.

Feed efficiency traits are moderately heritable, with estimates typically ranging from 0.1 to 0.4 depending on the specific trait definition and population studied (Brito et al., 2020b). The genetic control of feed efficiency is complex, involving multiple biological pathways related to digestion, metabolism, and energy use (Brito et al., 2020b; Jiang et al., 2024). One of the main challenges in improving feed efficiency through genetic selection is the difficulty and high cost of collecting accurate individual feed intake data on many animals. Traditional measurement approaches require specialized equipment and intensive management, which restricts the number of animals that can be evaluated. Recent technological advances are helping to overcome these barriers through the development of automated feed intake monitoring systems that employ electronic identification, load cells, and computer vision to continuously record individual animal feed consumption (Ojo et al., 2024). A promising feed intake recording system is the Cattle Feed Intake and Tracking (CFIT) system, which is an automated technology that uses 3-dimensional cameras and computer vision to continuously measure individual feed intake and feeding behavior of dairy cows under commercial conditions (Giagnoni et al., 2024). It provides accurate, noninvasive phenotyping at large scale, enabling genetic selection for feed efficiency and related traits. The integration of sensor technologies with feed intake measurements is creating new opportunities for genomic evaluations. Wearable devices can continuously monitor traits such as rumination, activity, and other behavioral indicators that may be associated with feed efficiency (Lopes et al., 2024). Combining these diverse data sources through machine learning approaches may improve the accuracy of efficiency predictions while reducing the cost of trait measurement (Shadpour et al., 2022).

Over the past years, there has been an increased global effort to reduce GHG emissions, particularly methane, including from livestock production. Dairy cattle alone account for ∼4% of anthropogenic GHG emissions worldwide (Singaravadivelan et al., 2023). In addition to being more feed efficient, the dairy cow of the future is expected to produce substantially less methane. Methane is a natural byproduct of rumen fermentation, where specific microorganisms convert hydrogen and carbon dioxide into methane as part of the normal digestive process. This process represents an energy loss of ∼6% to 12% of total energy intake, making methane reduction beneficial from both environmental and efficiency perspectives (Roche et al., 2018). There is substantial additive genetic variability in methane emission traits, with heritability estimates typically ranging from 0.15 to 0.35, depending on the trait definition and measurement method (Richardson et al., 2021; Kamalanathan et al., 2023). Various methane emission traits have been proposed, including total methane production, methane yield per unit of feed intake, and methane intensity per unit of milk production (Kamalanathan et al., 2023). The positive genetic relationship between methane production and milk yield (Pszczola et al., 2019; Rojas de Oliveira et al., 2024) suggests that selection for milk yield and fat content may inadvertently increase methane emissions, highlighting the importance of balanced breeding goals.

A major challenge for the genetic improvement of methane traits has been the difficulty and costs of accurately measuring individual emissions. Respiration chambers are accurate but costly and labor-intensive, which restricts their application in large-scale breeding programs. Recent technological advances have enabled the development of more scalable measurement approaches such as GreenFeed machines, laser methane detectors, and infrared sensors integrated into feeding systems or milking parlors (Ghassemi Nejad et al., 2024). Mid-infrared analysis of milk has also proven useful for predicting methane emissions with moderate accuracy and has been implemented in the national genetic evaluation schemes from Canada (Rojas de Oliveira et al., 2024). In addition, rumen microbial indicators, milk fatty acid profiles, and other biomarkers are under investigation as potential proxies for methane production. The genetic relationships between methane emissions and other economically important traits also indicate that genomic selection for reduced methane emissions without compromising performance is possible when appropriate trait definitions and selection strategies are applied (Kamalanathan et al., 2023; Rojas de Oliveira et al., 2024).

While incorporating an increasing number of traits in selection indexes enables more comprehensive and sustainable breeding objectives, it also dilutes the rate of genetic progress achieved for individual traits. According to selection index theory, when multiple traits are included with meaningful (bioeconomic) weights, the overall genetic gain is distributed across them in proportion to their relative importance and genetic correlations. Therefore, the inclusion of additional traits such as welfare, resilience, or environmental efficiency may reduce the short-term response in production traits but contributes to greater overall herd sustainability. Future breeding goals should thus be designed to achieve an optimal balance between productivity, animal well-being, and environmental impact rather than maximizing progress in any single trait.

International collaborations are essential for advancing genomic selection in dairy cattle, as they enable the pooling of large-scale data across countries, which can increase accuracy of breeding values and facilitate the development of robust models that are applicable to diverse populations (van Staaveren et al., 2024). Organizations such as the International Committee for Animal Recording (ICAR; https://www.icar.org) play a critical role in standardizing methodologies, fostering data exchange, and ensuring international comparability of evaluations, thereby strengthening global genetic improvement programs.

More recently, there has been an increasing control of data from sensors and other precision technologies by private companies or manufacturers, which limits farmer ownership and accessibility to raw phenotypic data. This shift has important implications for genetic improvement programs, as it may restrict the availability of large-scale, high-quality phenotypes that are essential for developing accurate genetic evaluations (Brito et al., 2025). Another highly relevant aspect of dairy cattle breeding, beyond breeding goals and phenotyping schemes, is the maintenance of genetic diversity both across and within breeds. Since domestication, hundreds of cattle breeds have been developed, but only a few dominate the dairy cattle sector, particularly the Holstein breed. In many countries, Holsteins represent ∼90% of the dairy cattle population and benefit from the largest dairy reference populations for genomic selection. For example, the CDCB genomic database includes 12 breeds, but 89% of the genotypes are from Holsteins and 10% from Jerseys (CDCB, 2025; https://uscdcb.com/database-stats/). Most novel traits are usually implemented first, and sometimes exclusively, in Holstein breeding programs, which further reinforces the breed's competitiveness. The large proportion of genotyped Holstein animals enables greater selection intensity; larger reference populations result in more accurate breeding values and, consequently, faster rates of genetic progress. For instance, Jerseys have traditionally been recognized for their high milk fat content. However, in recent years, the average breeding values of Holstein bulls have surpassed those of Jersey bulls, and Holstein cows now present values similar to Jersey bulls and higher than Jersey cows (Figure 2). Although many non-Holstein breeds possess unique advantages, the disproportionate availability of reference populations for novel traits, combined with the widespread adoption of reproductive tools and genomic technologies, may further increase Holstein's dominance. Without deliberate efforts at national and global levels to develop multicountry reference populations for smaller dairy breeds, and without strategies that ensure the long-term sustainability of dairy cattle breeds, across-breed genetic diversity may continue to erode.

**Figure 2:**
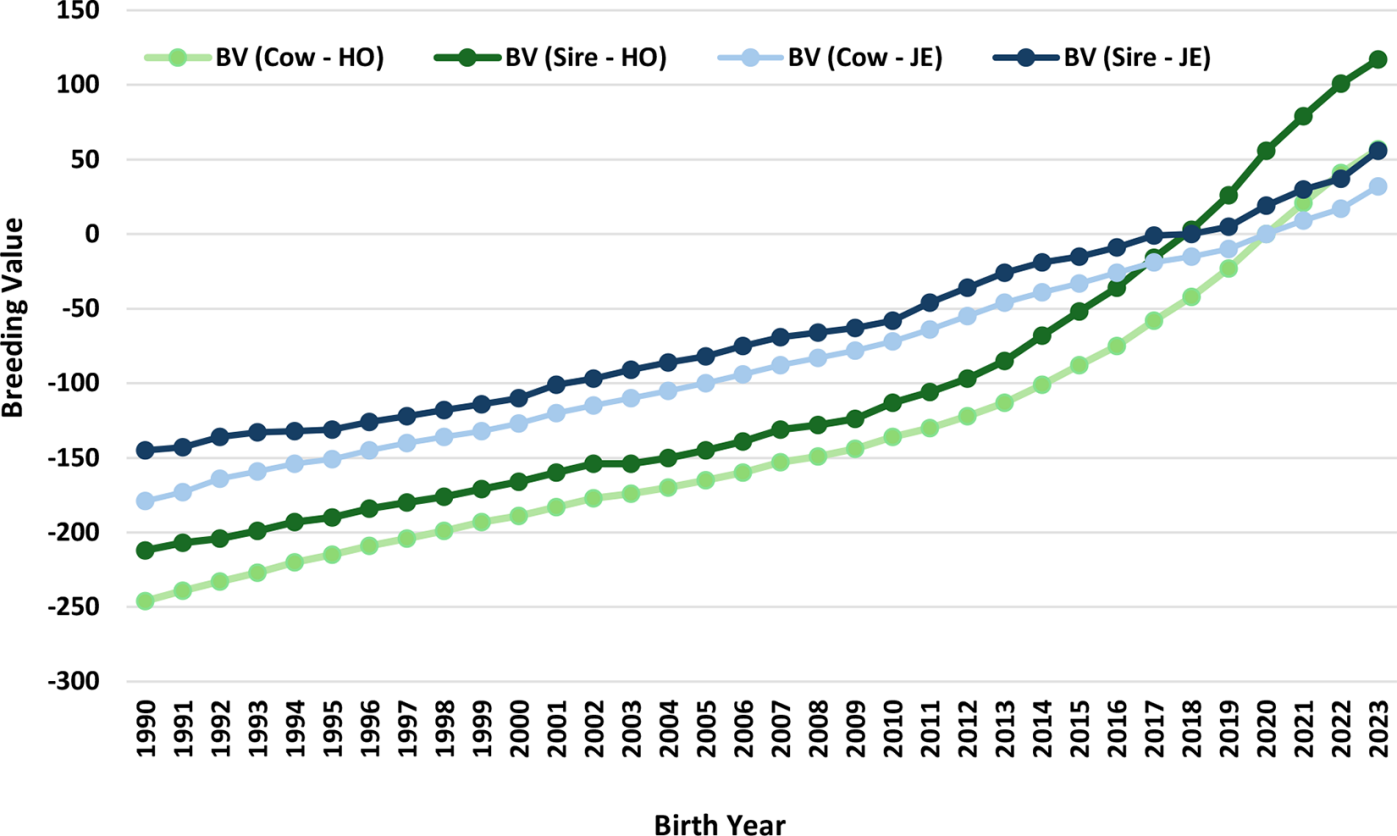
Genetic trends for milk fat content in US Holstein (HO) and Jersey (JE) cattle separated by cow and sire breeding values (BV). Source: Council on Dairy Cattle Breeding (CDCB, 2025b).

In addition to maintaining across-breed genetic diversity, within-breed genetic variability is of utmost importance. The annual rates of inbreeding in dairy cattle populations continue to increase (Brito et al., 2021; CDCB, 2025a), especially as generation intervals went down with the implementation of genomic selection (Guinan et al., 2023). Inbreeding is well known to have an unfavorable effect on livestock traits. For instance, based on a meta-analysis of 30 yr of research, Doekes et al. (2021) found that a 1% increase in pedigree inbreeding is associated with a median decrease in phenotypic value of 0.13% of a trait's mean and 0.59% of a trait's standard deviation. Inbreeding can also result in greater expression of alleles with unfavorable or deleterious effects in homozygous state. At the same time, genomic information can be used for more accurately assessing the relationship among animals, and therefore avoid mating of highly related animals, as well as more accurate inbreeding assessment based on metrics that separate ancient from more recent inbreeding (Meuwissen et al., 2020; Mulim et al., 2022). As more genomic data become available, more deleterious alleles can be detected in the populations, and used when doing selection and matings, as reviewed by Cole et al. (2025). A greater effort by all dairy sector stakeholders should be made to maintain within-breed genetic diversity and minimize the rates of inbreeding in dairy cattle populations. Meuwissen et al. (2020) describes various strategies for the management of genetic diversity in the genomics era. In brief, genomics data allow for calculation of many alternative measures of inbreeding and genomic relationships that can be used to manage genetic diversity in genomic optimal contribution selection schemes. Tools such as gene editing may also be used in the future for precisely removing deleterious or unfavorable alleles or adding genetic variability to targeted regions of the genome.

The dairy sector is experiencing a growing adoption of beef-on-dairy crossbreeding schemes (Berry, 2021), in which dairy cows (typically those with lower genetic merit) are inseminated with semen from beef bulls to produce offspring destined for the beef market. In contrast, dairy cows of higher genetic merit are inseminated with female sex-sorted semen to generate replacement heifers. Based on current market trends, it is expected that the vast majority (e.g., >90%) of dairy cows in the future will be inseminated with sex-sorted semen. In addition to such terminal crossbreeding strategies, dairy–dairy crossbreeding has also been widely adopted in some countries. For example, in New Zealand, the Kiwi Cross, resulting from crosses between Holstein and Jersey cattle, is a prominent system. Similarly, composite breeds and other crossbreeding schemes are popular in some countries. For instance, Girolando, a cross between Holstein and Gyr (*Bos taurus indicus*), is popular in Brazil due to its enhanced productivity and improved adaptation to hot and pasture-based production systems. From a crossbreeding perspective, selection pressure within dairy populations may increasingly favor cows that are more fertile, easy-calving, and capable of producing calves with acceptable growth and carcass traits when mated with beef sires, without compromising their own lifetime productivity and welfare.

There may also be some restructuring of the dairy sector in the coming decades. Various artificial insemination companies are developing contracts with dairy farmers for the collection of specific phenotypes of novel traits for providing customized genomic evaluations and also agreements for semen purchases and purchase of male calves to become breeding bulls. If continued, this may lead to a verticalization of the dairy breeding sector and the formation of subpopulations around artificial insemination companies. This could result in challenges for national and international genetic evaluations such as reduced connectedness among herds. Additional trends that may shape the future of dairy cattle breeding include the development of proprietary genomic evaluations by commercial companies, which may compete with and challenge national and international evaluation systems. More recently, there has been a shift toward isolationist policies and market restrictions (e.g., tariffs) in various countries and country blocks around the world, which threatens the international data collaboration essential for developing complex future traits.

In summary, we envision that dairy herds will exhibit reduced variability in the breeds represented, with a likely predominance of Holsteins, unless global initiatives are undertaken to preserve and enhance the sustainability of smaller breeds—an essential investment for the long-term sustainability of the dairy sector. The dairy cow of the future is expected to be healthier, more resilient, and more fertile, with improved adaptability to precision technologies such AMS. Future cows will be of more moderate size, demonstrate greater feed and environmental efficiency, produce higher levels of milk solids with finer composition, and live longer, while carrying fewer deleterious or unfavorable alleles.
